# Supplementary material for: Discharge domains regulation and dynamic processes of direct-current triboelectric nanogenerator
Source: Nat Commun. 2023 Jun 3;14:3218. doi: 10.1038/s41467-023-38815-9 (PMC10239477; doi:10.1038/s41467-023-38815-9)
Supplement: Supplementary file 1 — Supplementary Information [file 41467_2023_38815_MOESM1_ESM.pdf]

Supplementary Information

Discharge domains regulation and dynamic processes of direct-current triboelectric nanogenerator

Jiayue Zhang[1,δ], Yikui Gao[2,3,δ], Di Liu[2,3,δ], Jing-Shan Zhao[1,*], Jie Wang[2,3,*]

[1]Department of Mechanical Engineering, Tsinghua University, Beijing 100084, P. R. China.

[2]Beijing Institute of Nanoenergy and Nanosystems, Chinese Academy of Sciences, Beijing 101400, P.R. China.

[3]School of Nanoscience and Technology, University of Chinese Academy of Sciences, Beijing 100049, P.R. China.

[δ]J. Zhang, Yi. Gao and D. Liu contributed equally to this work.

*Corresponding Author: J.-S. Zhao: jingshanzhao@mail.tsinghua.edu.cn; J. Wang: wangjie@binn.cas.cn

Content

Supplementary Figures:

Supplementary Fig. 1 Paschen's curve: Gas is air at a pressure of 1 atmosphere.

Supplementary Fig. 2 Current output simulated by the single capacitor-breakdown model under different sliding speeds.

Supplementary Fig. 3 Combination of the capacitor-based simulation model and the random factor.

Supplementary Fig. 4 The cascaded-capacitor-breakdown model with multiple secondary capacitors.

Supplementary Fig. 5 Combination of two secondary capacitors.

Supplementary Fig. 6 Statistical result of the charge transferred between FE and CCE, which was normalized with the sliding area.

Supplementary Fig. 7 Strategy for determining the value of n.

Supplementary Fig. 8 Time-consuming under different values of n.

Supplementary Fig. 9 Sampling point selection principle.

Supplementary Fig. 10 Evidence for the 3rd breakdown occurs on the DC-TENG with end-isolated FE when the resistance in the external circuit is high.

Supplementary Fig. 11 Measurement configuration for the total triboelectric charge.

Supplementary Fig. 12 Measurement method for the residual surface charge.

Supplementary Fig. 13 Triboelectric charge densities collected by the DC-TENG with FE fully covered configuration.

Supplementary Fig. 14 Finite element method simulation results for the electric field intensity under the different vertical distances between FE and CCE.

Supplementary Fig. 15 Finite element method simulation results for the electric field intensity under the different horizontal distances between FE and CCE.

Supplementary Fig. 16 Finite element method simulation results for electric field intensities at specific positions in an open circuit.

Supplementary Tables:

Supplementary Table 1 Output power of conventional DC-TENG and the DC-TENG with end-isolated FE.

Supplementary Notes:

Supplementary Note 1 Working mechanism of a DC-TENG compared with a hydroelectric energy generating system.

Supplementary Note 2 Note on the surface charge.

Supplementary Note 3 Determination of the threshold of the electric field intensity base on Paschen's law.

Supplementary Note 4 Calculation for a single capacitor-breakdown model.

Supplementary Note 5 Analysis of the three main variables.

Supplementary Note 6 The random factor in the capacitor breakdown model.

Supplementary Note 7 The division strategy for secondary capacitors in the cascaded-capacitor-breakdown model.

Supplementary Note 8 Derivation for the breakdown threshold in charge of a single capacitor unit.

Supplementary Note 9 Determination of parameter n.

Supplementary Note 10 Calculation for root-mean-square (RMS) current and crest factor (CF).

Supplementary Note 11 Note on the examination of the variable σ_{CCE}.

Supplementary Note 12 Note on friction materials selection.

Supplementary Note 13 Sampling point selection principle.

Supplementary Note 14 Analysis of the evidence of electrostatic breakdown in electrical experimental data.

Supplementary Note 15 Measurement of the total triboelectric charges generated by the friction between FE and TL.

Supplementary Note 16 Measurement of the residual charges on the surface of TL after DC-TENG operation.

Supplementary Note 17 Influence of the isolation layer on contact electrification process and output signals.

Supplementary Note 18 Note on the equivalent circuit of DC-TENG.

Supplementary Note 19 Explanations for the 3rd electrostatic breakdown on the DC-TENG with end-isolated FE.

Supplementary Note 20 Influence of environmental conditions.

Supplementary Note 21 Generalization for the definition of discharge domains.

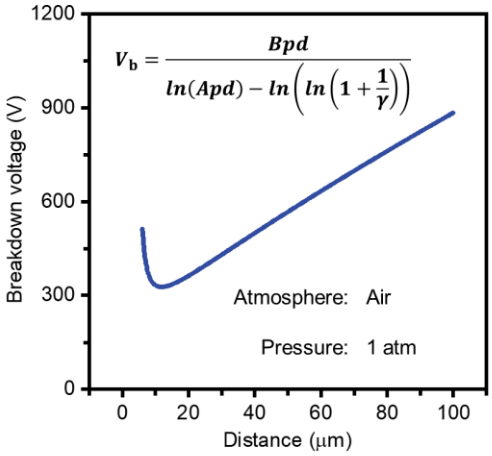

Supplementary Fig. 1 Paschen's curve: Gas is air at a pressure of 1 atmosphere. Source data are provided as a Source data file.

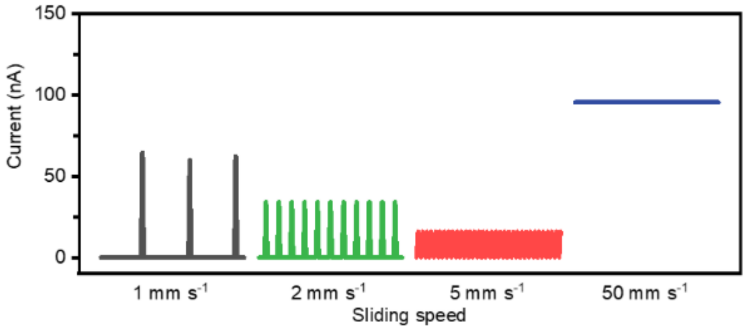

Supplementary Fig. 2 Current output simulated by the single capacitor-breakdown model under different sliding speeds. Source data are provided as a Source data file.

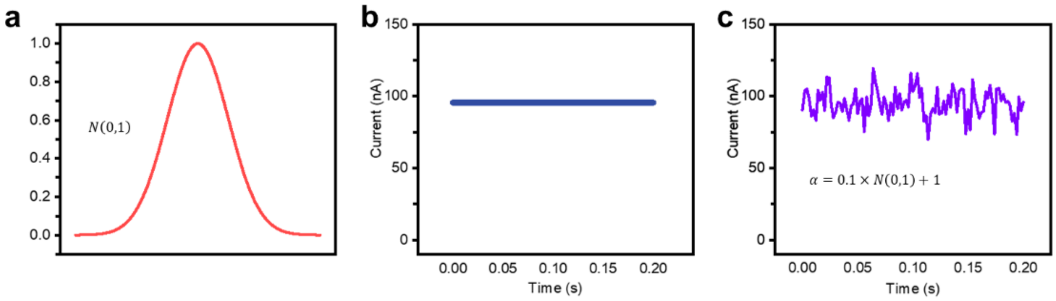

Supplementary Fig. 3 Combination of the capacitor-based simulation model and the random factor. a The normal distribution. **b** Constant input of the capacitor-breakdown simulation model. **c** Combining the input with the random factor. Source data are provided as a Source data file.

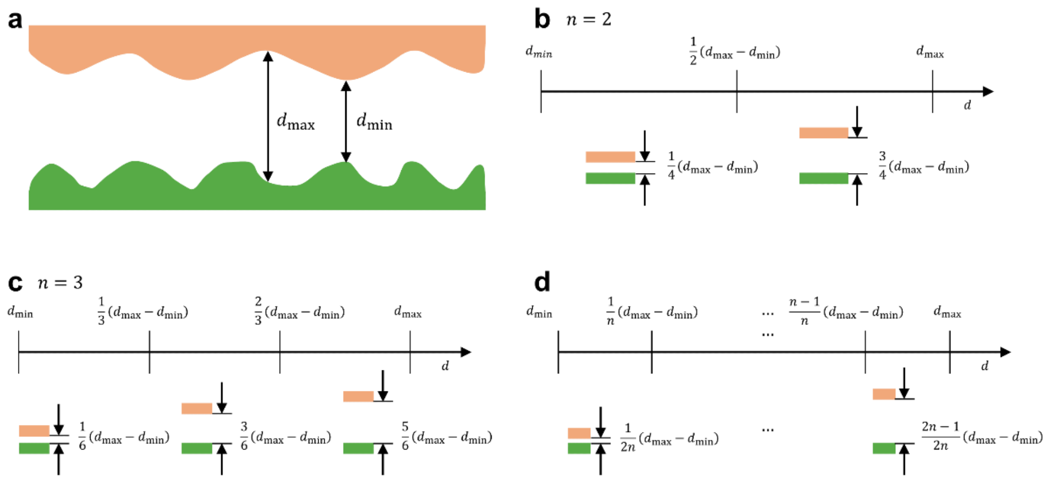

Supplementary Fig. 4 The cascaded-capacitor-breakdown model with multiple secondary capacitors. a Schematic diagram for the surface topography of charge collecting electrode (CCE) and triboelectric layer (TL) under microscopic view. Classification scheme when splitting the whole CCE-TL capacitor into **b** two, **c** three, and **d** n secondary capacitor models.

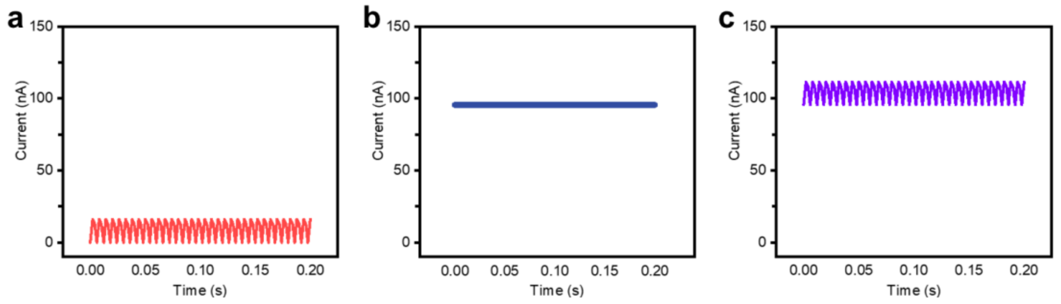

Supplementary Fig. 5 Combination of two secondary capacitors. a Simulated current output for a single secondary capacitor unit with pulsed outputs. **b** Simulated current output for a single secondary capacitor unit with constant-current outputs. **c** Combining the current outputs of the two secondary capacitors. Source data are provided as a Source data file.

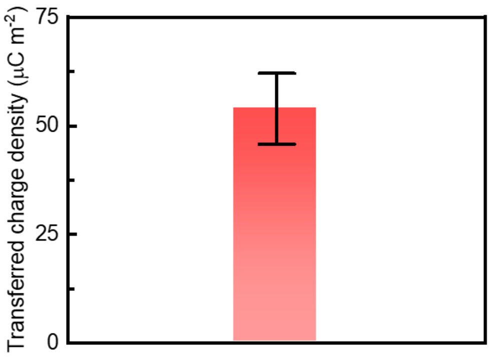

Supplementary Fig. 6 Statistical result of the charge transferred between FE and CCE, which was normalized with the sliding area. The error bar represents the standard deviation (six trials). Source data are provided as a Source data file.

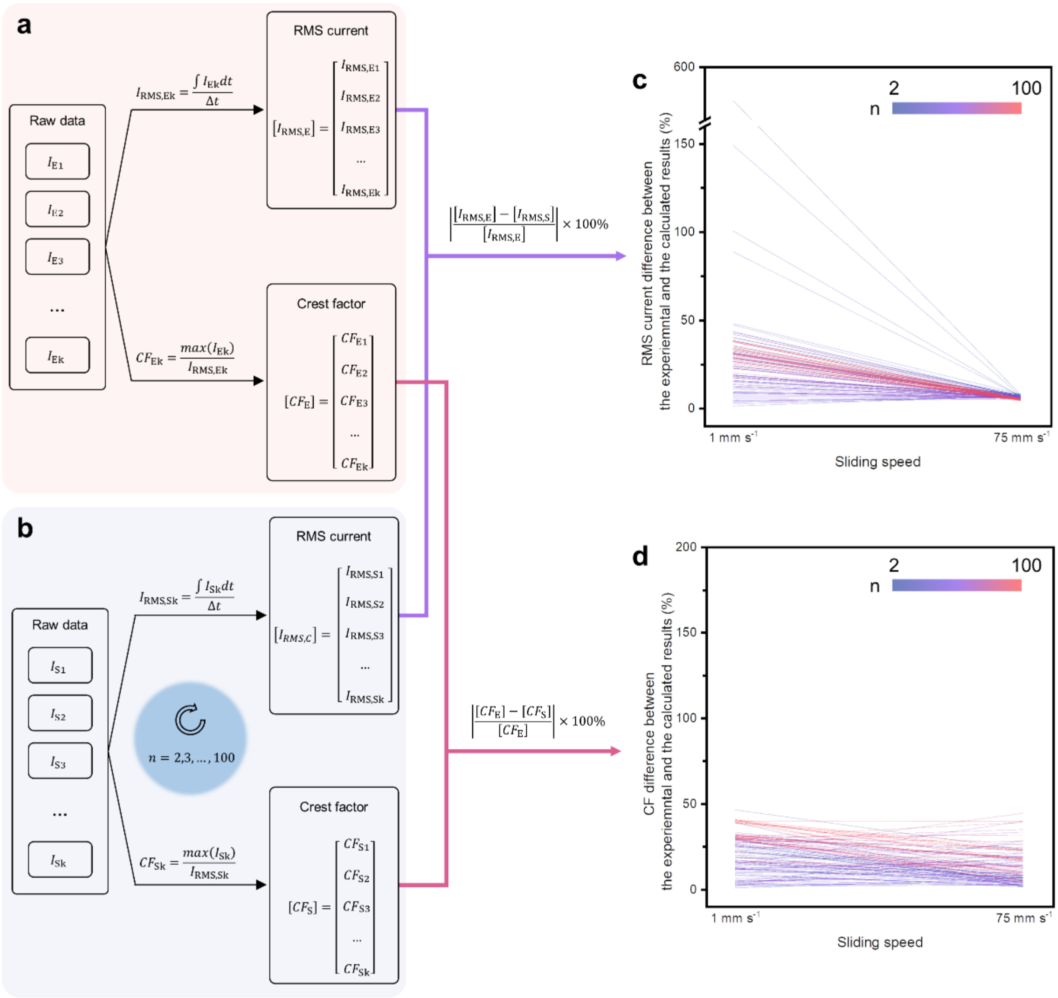

Supplementary Fig. 7 Strategy for determining the value of n. **a** Calculation of root-mean-square (RMS) current and crest factor (CF) for the experimental data. **b** The RMS current and CFs for the simulated results under different values of n, ranging from 2 to 100. The relative difference between the experimental data and simulated results on the evaluating parameter **c** The RMS current and **d** CFs with different values of n for the six speeds. Source data are provided as a Source data file.

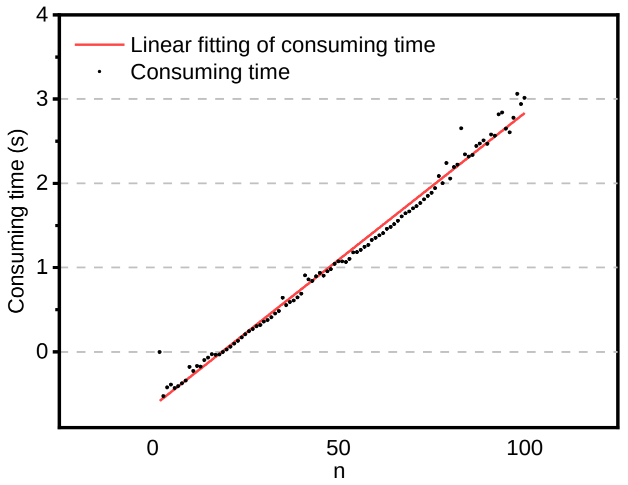

Supplementary Fig. 8 Time-consuming under different values of n. Source data are provided as a Source data file.

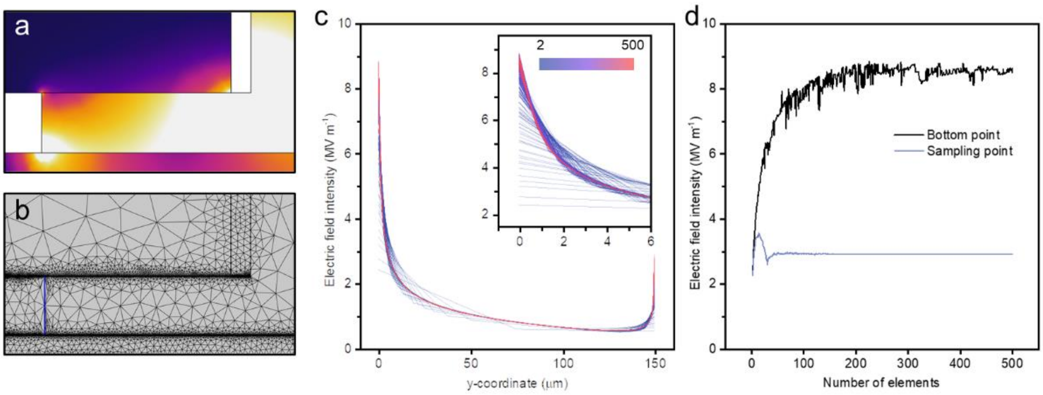

Supplementary Fig. 9 Sampling point selection principle. a Focusing region. **b** Mesh distribution in COMSOL. **c** Simulated electric field intensity with respect to the vertical coordinates on the edge of FE. **d** Simulated electric field intensity with respect to the number of elements on the edge of FE for the bottom point of FE edge and the sampling point 5 μm above it. Source data are provided as a Source data file.

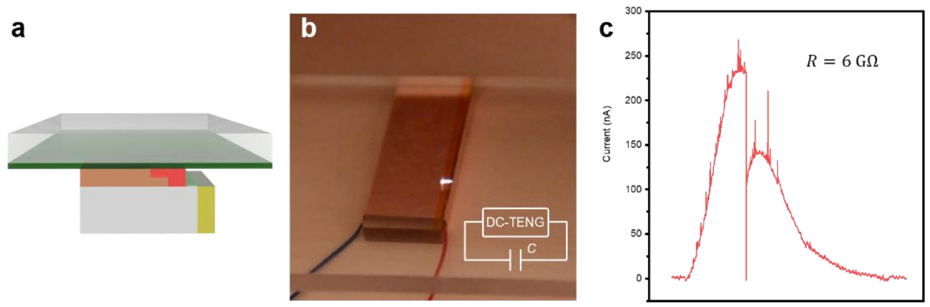

Supplementary Fig. 10 Evidence for the 3rd breakdown occurs on the DC-TENG with end-isolated FE when the resistance in the external circuit is high. a Configuration and hardware setting of the DC-TENG with end-isolated FE. **b** Photo for the discharge in the 3rd domain. **c** Breakdown signal on the current output. Source data are provided as a Source data file.

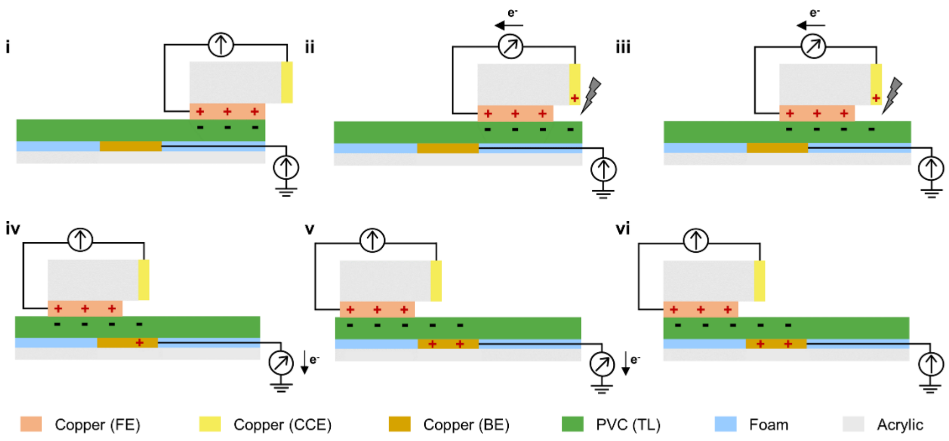

Supplementary Fig. 11 Measurement configuration for the total triboelectric charge.

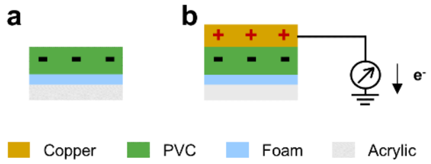

Supplementary Fig. 12 Measurement method for the residual surface charge. a TL surface after a DC-TENG device slides over. **b** An electrode is used to measure to the residual surface charge due to electrostatic induction.

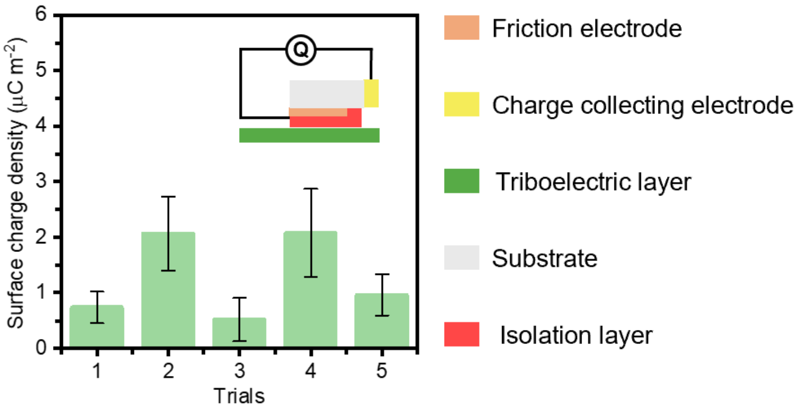

Supplementary Fig. 13 Triboelectric charge densities collected by the DC-TENG with FE fully covered configuration. Ten working cycles were measured for each trail. The error bar represents the standard deviation. Source data are provided as a Source data file.

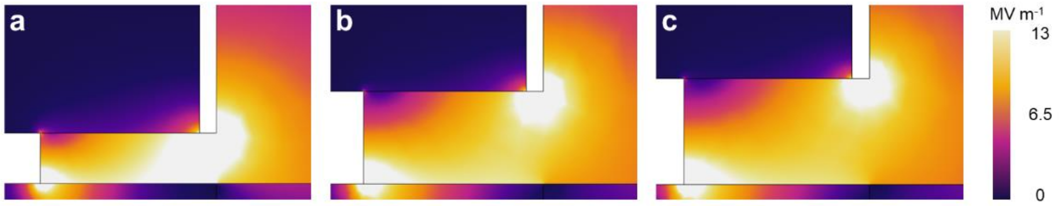

Supplementary Fig. 14 Finite element method simulation results for the electric field intensity under the different vertical distances between FE and CCE. The electric field intensity distribution with the vertical distance equals **a** 150, **b** 270, and **c** 300 μm.

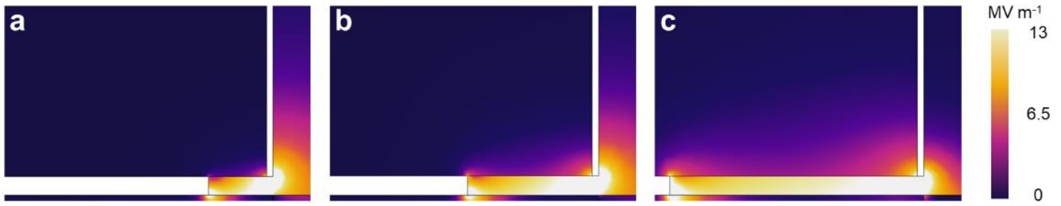

Supplementary Fig. 15 Finite element method simulation results for the electric field intensity under the different horizontal distances between FE and CCE. The electric field intensity distribution with the vertical distance equals to **a** 0.5, **b** 1.0, and **c** 2.0 mm.

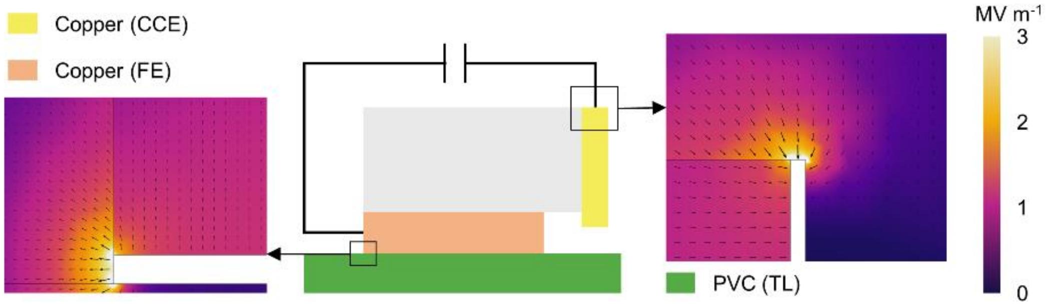

Supplementary Fig. 16 Finite element method simulation results for electric field intensities at specific positions in an open circuit.

Supplementary Table 1 Output power of conventional DC-TENG and the DC-TENG with end-isolated FE

Resistance (MΩ)	DC-TENG (μW)	DC-TENG with end-isolated FE (μW)	Improved ratio
1	0.0044	0.2126	47.32
10	0.0400	1.5227	37.07
100	0.4289	10.2106	22.81
200	0.9094	18.178	18.99
500	2.4843	38.3609	14.44
1,000	3.6741	65.4586	16.82
2,000	6.4460	96.5800	13.98
3,000	9.4047	128.2042	12.63

Supplementary Note 1 Working mechanism of a DC-TENG compared with a hydroelectric energy generating system

Compared with a hydroelectric energy generating system, FE, the external circuit, and CCE can be seen as a triboelectric power generating system. Based on the contact electrification effect, charges go into the triboelectric power generating system and leave the system due to the electrostatic breakdown phenomenon. The working path of those charges resembles that of a working fluid in a hydroelectric energy generating system (**Fig. 1a**). The work done on a hydroelectric system fills the container with fluid and inputs potential energy to the whole system, which is manifested in the DC-TENG system as friction between FE and the TL does work and generates charges into the system (**Fig. 1b(i)**). Under the gravity field, the water flows out and outputs power, for example, by driving the water wheel as demonstrated in **Fig. 1a**. Likewise, when the charged TL moves below CCE, the opposite charges are induced in CCE, resulting in a strong electric field between CCE and TL. Once the electric field intensity is higher than the breakdown threshold, the triboelectric charges on CCE and TL will discharge in the air[1], doing work in the external circuit (**Fig. 1b(ii)**). When the fluid level moves down below the outlet due to the decrease or stop of the inlet, the fluid will no longer do work to the outside. The identical condition occurs in the DC-TENG system, i.e., no current output after the relative motion stops (**Fig. 1b(iii)**). **Fig. 1b** demonstrates a working cycle, and the wire with a load in this diagram indicates the external circuit.

Supplementary Note 2 Note on the surface charge

As some previous work declared, the bipolar, multipolar, and mosaics of charges on tribo surfaces occur in micro and macro scales, especially for the contact between two dielectric layers.[2, 3] However, it is worth noting that these effects can be simplified to an equivalent charge, specifically the net charge, when applying contact electrification to TENG. This work focuses more on the output electrical performance of TENG, and the measured output signal is the result of the electric field generated by the superposition of all charges present in the entire area. In practice, regardless of how the local bipolar, multipolar, and mosaic patterns perform, the overall equivalent charge remains relatively stable. Hence, under this condition, for the sake of simplicity, the terms such as "charge", "surface charge density", "residual charge", and other similar expressions associated with electrostatic charges generated by contact electrification in this work refer to a simplified expression of "net charge", "net surface charge density", "net residual charge", and so forth.

Supplementary Note 3 Determination of the threshold of the electric field intensity base on Paschen's law

Paschen's law for gas breakdown gives an empirical relation between the breakdown voltage and the product of the gas pressure and the distance between the two electrodes involved in the breakdown process. The question is shown in **Equation S1.**

$$V_{\mathrm{b}} = \frac{Bpd}{\ln(Apd) - \ln[\ln\left(1+\frac{1}{\gamma}\right)]} \qquad \textbf{(S1)}$$

where V_{b} is the breakdown voltage; p is the gas pressure; d is the distance; A and B are constants determined by the pressure and composition of the gas; and γ is the secondary electron emission coefficient.

In our manuscript, the working condition was in the air of 1 atmosphere, so we plotted the Paschen's curve in **Supplementary Fig. 1** with A = 10.95 m^{-1} Pa^{-1}, B = 273.8 V m^{-1} Pa^{-1}, and $\gamma = 8.136\times10^{-3}$.

While researchers generally accept the validity of the Paschen's law for distances above 5-10 microns (i.e., the right side of the curve in **Supplementary Fig. 1**), there is ongoing debate regarding its applicability for smaller air gaps (i.e., the left side of the curve in **Supplementary Fig. 1**)[4-6], recently. In our work, we have to utilize the breakdown voltage at distances ranging from ~0 (the contact point of the friction electrode (FE) and the triboelectric layer (TL)) to millimeter scale (the distance between FE and charge collecting electrode (CCE)), which falls within the controversial region where the accuracy of Paschen's law is debated. We derived the breakdown threshold of the electric field intensity from the calculated breakdown voltage, which varied from 3 MV m^{-1} to 7 MV m^{-1} for the right side of Paschen's curve. The DC-TENG device itself presents many non-ideal factors that further complicate the analysis of its electrical properties. For instance, the electric field around the DC-TENG is not uniform as assumed in Paschen's law, which is probably due to the mechanical configuration, materials selections, and electromagnetic environment issues. To simplify our model and emphasize the main focus of this work, we used a breakdown voltage magnitude that many researchers have employed, which is 3 MV m^{-1} [5, 7-9].

For the left side of the curve, some works stated that in addition to the Townsend avalanche of gaseous ions, other effects such as field emission of electrons and tunneling electrons have been proposed as potential factors, and they offered a modified Paschen's curve with a slope of 60-80 MV m^{-1} [4-6]. Whether considering the original Paschen's curve or the modified one, the breakdown electric field intensity exceeds 3 MV m^{-1} by more than an order of magnitude, which does not align with real-world conditions. For instance, the electric field intensity threshold for triggering the field emission is ~75 MV m^{-1} [5], requiring ultra-high surface charge density of ~1330 μC m^{-2}, which is a strict condition to achieve in the air with 1 atmosphere. Hence, 3 MV m^{-1} can be a proper magnitude of breakdown threshold.

Supplementary Note 4 Calculation for a single capacitor-breakdown model

Before the electrostatic breakdown occurs, the focusing region at CCE and TL can be treated as a parallel capacitor. The equation for calculating the capacitance is shown as following:

$$C_{\mathrm{CCE-TL}} = \frac{\varepsilon S}{d} = \varepsilon \frac{L_{\mathrm{CCE}} w_{\mathrm{CCE}}}{d_{\mathrm{CCE-TL}}} \tag{S2}$$

where ε denotes the permittivity of the material, which is air in this application, between CCE and TL, S denotes facing area of a parallel capacitor, d denotes the vertical distance between the two parallel plates, L_{CCE} denotes the length of CCE in the direction along the sliding direction, w_{CCE} denotes the width of CCE perpendicular the sliding direction, and $d_{\mathrm{CCE-TL}}$ denotes the vertical distance between CCE and TL. Further, the voltage between CCE and TL $U_{\mathrm{CCE-TL}}$ can be calculated with the charge in CCE and TL $Q_{\mathrm{CCE-TL}}$:

$$U_{\mathrm{CCE-TL}} = \frac{Q_{\mathrm{CCE-TL}}}{C_{\mathrm{CCE-TL}}} \tag{S3}$$

Calculated from the voltage, the electric field intensity between CCE and TL is

$$E_{\mathrm{CCE-TL}} = \frac{U_{\mathrm{CCE-TL}}}{d_{\mathrm{CCE-TL}}} \tag{S4}$$

When $E_{\mathrm{CCE-TL}}$ is larger than the breakdown threshold in the air, which is 3 MV m^{-1}, the focusing region at CCE and TL can be seen as a resistor $R_{\mathrm{CCE-TL}}$. Then the output current can be obtained as

$$I_{\mathrm{CCE-TL}} = \frac{U_{\mathrm{CCE-TL}}}{R_{\mathrm{CCE-TL}}} \tag{S5}$$

Taking the sliding speed as an example variable and keeping other variables fixed, calculated by the single capacitor-breakdown model, low sliding speed leads to pulse signals (**Supplementary Fig. 2a**). As the input increases, the frequency of the pulse increases (**Supplementary Fig. 2b** and **2c**), and when the input increases to a specific value, a constant current output is obtained (**Supplementary Fig. 2d**).

Supplementary Note 5 Analysis of the three main variables

Charges inlet on CCE Q_{CCE} can be formulated as a product of the surface charge density on TL σ_{CCE} and the swept area $S_{\mathrm{CCE-TL}}$.

$$Q_{\mathrm{CCE}} = \sigma_{\mathrm{CCE}} S_{\mathrm{CCE-TL}} \tag{S6}$$

Since the production of the charge is continuous as FE keeps sliding on TL, the rate of charge inlet is the derivative of equation S6.

$$\dot{Q}_{\mathrm{CCE}} = \frac{\mathrm{d}\sigma_{\mathrm{CCE}} S_{\mathrm{CCE-TL}}}{\mathrm{d}t} = \sigma_{\mathrm{CCE}} w_{\mathrm{CCE}} \frac{\mathrm{d}x_{\mathrm{CCE-TL}}}{\mathrm{d}t} \tag{S7}$$

where $x_{\mathrm{CCE-TL}}$ is the relative displacement of CCE and TL, and the derivative of it is thus the sliding speed v_{sliding}. Hence, the inlet rate of charges on $C_{\mathrm{CCE-TL}}$ can be modeled by the following equation.

$$\dot{Q}_{\mathrm{CCE}} = \sigma_{\mathrm{CCE}} w_{\mathrm{CCE}} v_{\mathrm{sliding}} \tag{S8}$$

Supplementary Note 6 The random factor in the capacitor breakdown model

The roughness of TL can affect contact electrification performance by altering the real area of contact and contact pressure. Considering the charge generation side, a random factor α is employed to take practically inconsistent conditions into account, such as the uniformity of materials, the roughness of two contacting surfaces, and contact efficiency. It conforms to the normal distribution (**Supplementary Fig. 3a**). With this random factor, the rate of charge flowing into the capacitor is not a constant (**Supplementary Fig. 3b**), but a value with kind of randomness (**Supplementary Fig. 3c**), which is consistent with the practical conditions.

Supplementary Note 7 The division strategy for secondary capacitors in the cascaded-capacitor-breakdown model

In this cascaded-capacitor-breakdown model, the roughness also influences the electrostatic breakdown phenomena. The roughness of the TL can make the distance between the TL surface and the bottom surface of the CCE inconstant. According to Paschen's law, different air gaps lead to different breakdown characteristics. Therefore, to reflect real-world conditions, the cascaded-capacitor-breakdown model occupies multiple secondary capacitor units. When it comes to the discharge process, the capacitor composed of CCE and TL is further divided into several secondary capacitor units according to the real distance between CCE and TL, since the two facing surfaces of CCE and TL are not perfectly flat from microscopic view due to the practical factors, such as material properties and fabrication process (**Supplementary Fig. 4a**). To be more specific, by dividing the whole focusing region between CCE and TL into two categories due to the statistical analysis in the microscopic view, the area with the distance of $d_{\min}$ to $\frac{(d_{\max}-d_{\min})}{2}$ is divided into one category, and that of $\frac{(d_{\max}-d_{\min})}{2}$ to $d_{\max}$ is divided into another category (**Supplementary Fig. 4b**). Similarly, **Supplementary Fig. 4c** and **Supplementary Fig. 4d** illustrate the situation when more categories are classified.

Supplementary Note 8 Derivation for the breakdown threshold in charge of a single capacitor unit

Assuming each capacitor unit is a parallel capacitor, combining equations S2, S3, and S4, the electric field intensity between each capacitor unit E_{unit} is derived as

$$E_{\text{unit}} = \frac{Q_{\text{unit}}}{\varepsilon S_{\text{unit}}} \tag{S9}$$

where Q_{unit} is the charge on both sides of the parallel capacitor unit and S_{unit} is the facing area of a single parallel capacitor unit, determined by the number of the parts the whole capacitor is divided into.

From equation S9, electric field intensity is irrelated to the vertical distance between the two parallel plates. Hence all the capacitor units have the same breakdown threshold. Further, the judgment threshold in charge shown in **Fig. 1d** can be calculated as

$$Q_{\text{threshold}} = \varepsilon S_{\text{unit}} E_{\text{threshold}} \tag{S10}$$

The breakdown threshold for capacitor units with different vertical separation distances is the same. After the breakdown threshold is reached, the output current for a single capacitor unit can be calculated as

$$I_{\text{unit}} = \frac{E_{\text{unit}} d_{\text{unit}}}{R_{\text{unit}}} \tag{S11}$$

Since the secondary capacitors are divided by the real-world distance between CCE and TL, the after-breakdown resistance for each secondary capacitor varies.

Supplementary Note 9 Determination of parameter *n*

As some parameters in the cascaded-capacitor-breakdown simulation model require pre-experimental calibration, experimental data at two velocities (1 mm s^{-1} and 75 mm s^{-1}) was used as the tuning set, while experimental data at four velocities (5, 10, 25, and 50 mm s^{-1}) is used as the test set. σ_{CCE} was determined by pre-experiments (**Supplementary Fig. 6**). The number of secondary capacitors n was determined by comparing the simulated results and the tuning set experimental data. A root-mean-square (RMS) current matrix with the dimension of 2×1 and a crest factor (CF) matrix with same size has been calculated for each experimental group. The definition and calculation of the RMS current and CF are shown in **Supplementary Note 9**. The dimension of the RMS current matrix indicates experimental data at two velocities (1 mm s^{-1} and 75 mm s^{-1}) was used as the tuning set. Then, traversed from 2 to 100, for each value of n, a set of the RMS current matrix and CF matrix was generated. The relative differences between the simulated matrixes and the experimental matrixes on the key evaluation factors the RMS currents and CFs for each n value was calculated (**Supplementary Fig. 7a** and **7b**, where the subscript "E" represents experimental data, the subscript "S" represents the simulated results, and the subscript "k" indicates the dimension of the tuning set). The general trend is the difference between the experimental data and the simulated results decreases with an increasing n (**Supplementary Fig. 7c** and **7d**). Nevertheless, n cannot be infinitely large, since the simulation time is proportional to the value of n and the computing resources are finite (**Supplementary Fig. 8**). Comprehensively considering the statement above, $n = 35$ was chosen for this experiment. For future applications, with more data, the determination of parameter n can be modified by increasing the tuning set size (the value of k) and the range of n, as shown in **Supplementary Fig. 7a** and **Supplementary Fig. 7b**.

Supplementary Note 10 Calculation for root-mean-square (RMS) current and crest factor (CF)

The root-mean-square (RMS) current mentioned in this work is defined as the value of the constant current that dissipates the same power in a resistor as the time-varying current does by our DC-TENG, which can also be called effective current. The equal derived from the energy equation is shown as follows:

$$\int_0^T I(t)^2 R dt = I_{\mathrm{RMS}}^2 RT \quad \text{(S12)}$$

$$I_{\mathrm{RMS}} = \frac{\int_0^T I(t)^2 dt}{T} \quad \text{(S13)}$$

where $I(t)$ is a set of current data measured within time T.

The crest factor (CF) mentioned in this work is defined as the ratio of peak current to the effective current, indicating how extreme the peak value is in a current waveform. A CF of 1 suggests no peaks in the current signals, indicating a constant-current output. The equation used in this work is

$$CF = \frac{I_{\max}}{I_{\mathrm{RMS}}} \quad \text{(S14)}$$

where $I_{\max}$is the maximum value in a set of current data.

Supplementary Note 11 Note on the examination of the variable σ_{CCE}

Variable σ_{CCE} is obtained by measuring the charges collected at CCE, and dividing this value with the area swept by the relative motion. It is mainly influenced by charging and discharging processes of $C_{\mathrm{CCE-TL}}$. In the charging process, part of the charges generated by the friction between FE and TL flows into this equivalent capacitor. In the discharging process, these charges participate the electrostatic breakdown at CCE. Hence, σ_{CCE} is constrained by the contact electrification and electrostatic breakdown properties of the material-pairs[10]. In this work, both contact electrification and electrostatic breakdown are achieved with a copper electrode and a dielectric layer. Therefore, we kept the electrode unchanged and altered the material of the dielectric layer to regulate σ_{CCE}.

Supplementary Note 12 Note on friction materials selection

It is worth noting that when selecting friction material-pairs, in addition to considering the surface charge density that the material-pair can generate, other properties of the material[10], such as the friction coefficient, manufacturing process, and the sensitivity to temperature and humidity should also be considered. In the experiment, it was found that the surface charge density generated by the friction between ethylene tetrafluoroethylene (ETFE) and the copper electrode was high, but the large friction coefficient caused it difficult for FE to smoothly slide on TL. This caused the low contact efficiency and an unstable contact electrification process at low speed, resulting in a fluctuating output current. Besides, it puts unpredictable stress on the device, leading to wear and tear and shortening the life of devices.

Supplementary Note 13 Sampling point selection principle

When discussing the electric field intensity distribution in the 1st and 2nd domains, two sampling points were used to represent their situation. The electric field intensity is generally strongest at sharp corners, making them the most likely location for discharge to occur. However, in finite element method simulations, the calculation of the sudden change in the geometry is highly dependent on the mesh distribution, and also turns out unreliable due to geometric discontinuity. Focusing on the region we were interested in, as shown in **Supplementary Fig. 9a**, the mesh distribution was displayed in **Supplementary Fig. 9b**. The sampling point in the 2nd domain was taken as an example to declare the reason for selecting such sampling points in finite element method simulations in the manuscript. By changing the element number of the edge of FE, which was highlighted in **Supplementary Fig. 9b**, the electric field intensity on each gridding point was simulated and shown in **Supplementary Fig. 9c**. The electric field intensities at the two ends are larger than that of the gridding points in the middle and the fluctuation with the number of elements are also greatest on these two positions (inset in **Supplementary Fig. 9c**). To ensure consistent simulation results and minimize the impact of the number of elements on the outcome, we selected points that were not at the two ends but close to where the maximum electric field intensity occurs as sampling points. Although the electric field intensities at these points is relatively low (**Supplementary Fig. 9d**), it has no influence on our determination of whether breakdown will occur in the 1st and 2nd domains. When the electric field intensity at a sampling point exceeds the breakdown threshold, there must be a point with an even higher electric field intensity in the corresponding discharge domain, indicating the breakdown will definitely occur. Selecting the sampling point a short distance away from a geometrically sharp point allows for more stable and reliable numerical simulation (**Supplementary Fig. 9d**). The discarded magnitude of this part of electric field intensity further supports the existence of the two breakdown domains we defined.

Supplementary Note 14 Analysis of the evidence of electrostatic breakdown in electrical experimental data

In a short circuit, while measuring the current data in **Fig. 2**, the total amount of triboelectric charge and the surface residual charge after the electrostatic breakdown were measured at various speeds using the method described in **Supplementary Fig. 11, 12** and **Supplementary Note 15, 16**. As shown in **Fig. 4k**, comparing the total triboelectric charge with the charge collected in the external circuit, it is found that a large amount of charge did not flow through the external circuit. This part of the charge is prone to release from 2nd domain through an electrostatic breakdown in the air.

In an open circuit, with relative motions, the triboelectric charges concentrated on FE lead to a significant potential difference between FE and CCE, equivalent to applying a bias voltage between the two[11]. On the one hand, this potential difference will make it difficult for triboelectric charges to enter or remain in FE. As shown in **Fig. 4l**, when charging a test capacitor with DC-TENG as shown in the circuit inset, as the number of working cycle increases, the amount of charge that can be charged into the test capacitor in a single cycle decrease. On the other hand, considering FE and CCE as a capacitor, the voltage between the two electrodes is proportional to the amount of charge it contains. When the charge accumulated to a certain amount, the breakdown voltage was reached, resulting in the electrostatic breakdown in the 3rd domain and charges being released, as marked with the pentagram in **Fig. 4k**.

Supplementary Note 15 Measurement of the total triboelectric charges generated by the friction between FE and TL

As shown in **Supplementary Fig. 11(i-iii)**, with relative sliding between FE and TL, the triboelectric charge is generated due to contact electrification and discharged due to electrostatic breakdown. When the DC-TENG device moves onto the back electrode, the discharge is electrostatically shielded since there is charge induced on the back electrode. By measuring the amount of charge transferred from the ground to the back electrode, the equal charge generated on the TL via contact electrification can be thus measured. From another perspective, the back electrode, dielectric layer, and the sliding FE can be seen as a single electrode sliding mode alternating current TENG (AC-TENG)[12], and the amount of charge on the dielectric layer can be determined by measuring the transferred charge in the external circuit.

Supplementary Note 16 Measurement of the residual charges on the surface of TL after DC-TENG operation

After DC-TENG stops, an electrode connected to the ground was put onto the surface of TL. Due to electrostatic induction, positive charges will flow from the ground to the contact surface of the electrode to balance the potential difference. By measuring the amount of these induced charges, the residual surface charge can be measured. The residual surface charge density was obtained by dividing the residual surface charge with the area of the testing electrode, which is 1 cm^2 for the experiments with different widths of CCE, and 4 cm^2 for other experiments in this paper.

Supplementary Note 17 Influence of the isolation layer on contact electrification process and output signals

Although there is contact electrification effect between the Kapton tape and the PVC film, it has little influence on the output performance of the DC-TENG. Kapton is a kind of dielectric material, and after a brief rubbing, the charge on the Kapton tape becomes saturated. Since the conductive electrode on the other side of the Kapton tape serves as a back electrode to bound charges[13], the discharge between Kapton and PVC rarely occurs. Hence, the saturated Kapton no longer participates in the contact electrification process, simply acting as an isolating tool with ignorable influence on the electric field or electric output. As shown in **Supplementary Fig. 13**, compared with the charge density measured in the external circuit of DC-TENG with uncovered FE (dozens of micro coulombs per square meter), that of the DC-TENG with covered FE configuration (inset of **Supplementary Fig. 13**) can be negligible (~1.3 $\mu C\ m^{-2}$).

Supplementary Note 18 Note on the equivalent circuit of DC-TENG

To better understand it in a circuit, an equivalent circuit is shown in **Fig. 6b**. The electric properties in the 1st, 2nd, and 3rd domain are similar, so FE and CCE, FE and TL can also be analyzed as capacitor models similar to the one in **Fig. 1c**. Based on relative position of these mechanical structure, the equivalent circuit was constructed. The current source represents the continuous inlet charges of the triboelectric system due to contact electrification. Together with generation, some charges are released through the 2nd BD. Equivalent secondary circuits of the 1st BD and 2nd BD are connected in parallel while the secondary circuit of the 3rd BD is in series to the 1st BD. As the circuit presented, once the 3rd breakdown releases all the charges in the system, there is no electrical output in the external circuit, though it rarely happens.

Supplementary Note 19 Explanations for the 3rd electrostatic breakdown on the DC-TENG with end-isolated FE

It is worth noting that as the resistance in the external circuit increases, it is easier to observe the 3rd BD for the DC-TENG with end-isolated FE compared to the conventional configuration. Visible evidence is taken with the same setting to **Fig. 4h**, as shown in **Supplementary Fig. 10a**. The bright discharge path connecting the two edges of the Kapton tape displays in **Supplementary Fig. 10b** proves that, on the one hand, the tape effectively protected FE from being exposed to the air, and on the other hand, the amount of charge accumulated in FE was so large that the breakdown in the 3rd domain was triggered. **Supplementary Fig. 10c** provides the output current under a resistance of 6 GΩ, obviously showing the 3rd BD. A possible explanation is that, with the 2nd domain blocked, some charges that originally flowed out from the 2nd domain leaves the triboelectric system from 1st domain, and the rest stays in the system and accumulates until the breakdown threshold of the 3rd BD is reached. Moreover, some tiny spikes on the current signals might be caused by the discharge between TL and the small strip of tape without metal electrodes on the back. Further modifications to the fabrication are required to solve this issue.

Supplementary Note 20 Influence of environmental conditions

For the sake of controlling variables and simplifying the theoretical model and experiments to a certain extent, consistent environmental parameters were employed throughout this work. However, it is worth noting that in practical applications, both temperature and relative humidity can impact the operation of DC-TENG. The presence of adsorbed water on the tribo surfaces is crucial for both the charging and discharging processes[14]. Its role in the triboelectrification process depends on the motion mode (contact-separation mode, sliding or free-standing mode), materials (chemical and physical properties characterized by the contact angle, functional group, roughness, and inherent triboelectrification ability), and other operational parameters. For discharging process, humidity may alter the breakdown characteristics of the gas, resulting in a noticeable decrease in the breakdown voltage as humidity increases[15]. As the DC-TENG device arises from both triboelectrification and electrostatic discharge, its output performance is influenced by both physical processes. Some research suggested that the output performance of DC-TENG is improved in high-humidity environments, as a high-humidity environment not only promotes the electrification effect of the hydrophobic materials to generate more triboelectric charges but also reduces the gap breakdown voltage, facilitating the electrostatic breakdown process. Nevertheless, the complex influence of humidity on the two physical processes is still controversial, and its influence on the output performance of DC-TENG needs more comprehensive study.

Supplementary Note 21 Generalization for the definition of discharge domains

To better explain and further utilize electrostatic breakdowns occurring in this triboelectric power generation, we generalized and presented more explanations about the concept of discharge domain for a conventional DC-TENG. In addition to the domains defined above, electrostatic breakdown may also occur at other locations due to manufacturing process, materials selection, and geometry design, especially where there is a sharp corner geometry (**Supplementary Fig. 16**).

These locations can also be classified into the categories defined above. The discharge participated by the conductor between the bottom of CCE and the electrical appliance in the external circuit can be classified into the breakdown in the 1st domain since it has a positive impact on the output of a conventional DC-TENG. It is because whichever the discharge on CCE or the wire, the negative charge tends to flow to FE to balance the potential difference continuously generated by contact electrification, which causes the charge to flow through the external circuit and does work externally. The discharge participated by the conductor between the contact electrification interface of FE and the electrical appliance in the external circuit can be classified into the breakdown in the 2nd domain since it has a negative effect. The reason is both the generation of the whole triboelectric charges and the release of charges in the 2nd domain happens before the electrical appliance, which means the amount of charge that flows through the electrical appliance is the net charge that equals the total triboelectric charge minus the charge released by 2nd breakdown, much less than the original triboelectric charge, restricting the output performance.

Reference

1. S. Wang, et al. Maximum surface charge density for triboelectric nanogenerators achieved by ionized-air injection: Methodology and theoretical understanding. *Adv. Mater.* **26,** 6720-6728 (2014).
2. H. Baytekin, et al. The mosaic of surface charge in contact electrification. *Science* **333,** 308-312 (2011).
3. Y. I. Sobolev, W. Adamkiewicz, M. Siek & B. A. Grzybowski Charge mosaics on contact-electrified dielectrics result from polarity-inverting discharges. *Nat. Phys.* **18,** 1347-1355 (2022).
4. D. B. Go & D. A. Pohlman A mathematical model of the modified Paschen's curve for breakdown in microscale gaps. *J. Appl. Phys.* **107,** 103303 (2010).
5. A. J. Wallash & L. Levit Electrical breakdown and ESD phenomena for devices with nanometer-to-micron gaps. *Proc. SPIE* **4980,** 87-96 (2003).
6. A. Peschot, N. Bonifaci, O. Lesaint, C. Valadares & C. Poulain Deviations from the Paschen's law at short gap distances from 100 nm to 10 μm in air and nitrogen. *Appl. Phys. Lett.* **105,** 123109 (2014).
7. D. Liu, et al. A constant current triboelectric nanogenerator arising from electrostatic breakdown. *Sci. Adv.* **5,** eaav6437 (2019).
8. D. Liu, L. Zhou, Z. L. Wang & J. Wang Triboelectric nanogenerator: From alternating current to direct current. *iScience* **24,** 102018 (2021).
9. Z. Zhao, D. Liu, Y. Li, Z. L. Wang & J. Wang Direct-current triboelectric nanogenerator based on electrostatic breakdown effect. *Nano Energy* 107745 (2022).
10. Z. Zhao, et al. Selection rules of triboelectric materials for direct-current triboelectric nanogenerator. *Nat. Commun.* **12,** 1-8 (2021).
11. Y. S. Zhou, et al. Manipulating nanoscale contact electrification by an applied electric field. *Nano Lett.* **14,** 1567-1572 (2014).
12. S. Wang, L. Lin & Z. L. Wang Triboelectric nanogenerators as self-powered active sensors. *Nano Energy* **11,** 436-462 (2015).
13. W. He, et al. Boosting output performance of sliding mode triboelectric nanogenerator by charge space-accumulation effect. *Nat. Commun.* **11,** 1-8 (2020).
14. L. Liu, et al. A high humidity-resistive triboelectric nanogenerator via coupling of dielectric material selection and surface-charge engineering. *J. Mater. Chem. A* **9,** 21357-21365 (2021).
15. L. Liu, et al. Achieving ultrahigh effective surface charge density of direct-current triboelectric nanogenerator in high humidity. *Small* **18,** 2201402 (2022).
